# Correlation of cord blood telomere length with birth weight

**DOI:** 10.1186/s13104-017-2791-6

**Published:** 2017-09-08

**Authors:** Siew-Peng Lee, Prakash Hande, George SH Yeo, Ene-Choo Tan

**Affiliations:** 1 Research Laboratory, KK Women’s and Children’s Hospital, 100 Bukit Timah Road, Singapore, Singapore; 2 Department of Physiology, Yong Loo Lin School of Medicine, National University of Singapore, Singapore, Singapore; 3 Maternal–Fetal Medicine, KK Women’s and Children’s Hospital, Singapore, Singapore; 4 Paediatrics Academic Clinical Programme, SingHealth Duke-NUS Medical School, Singapore, Singapore

**Keywords:** Birth weight, Newborns, Quantitative PCR, Telomere length

## Abstract

**Background:**

Intrauterine growth restriction affects 3% of newborns; and the lightest 10% of whom are classified as small for gestational age (SGA). These low-birth weight newborns are at increased risk of neonatal morbidity such as hypoxia and hypoglycaemia. In later life, they are at higher risk of several age-related diseases such as cardiovascular and metabolic disorders and dementia. As having short telomeres is also associated with these diseases, we tested if these newborns might already start with shorter telomeres at birth.

**Findings:**

Relative telomere lengths were determined using quantitative real-time PCR in cord blood samples from 195 newborns of Chinese ancestry. Based on the telomere length normalised to a single copy gene and a reference DNA sample as internal control, we found statistically significant correlations between relative telomere length and both unadjusted and gestational age-adjusted birth weight, with the lighter newborns having shorter telomeres. The SGA birth weight group comprising the bottom 10% of the samples also had the shortest telomeres compared to the medium and heaviest birth weight groups.

**Conclusions:**

Our results indicate that there is reduction of cord blood telomere length for newborns with lower birth weight.

## Background

Telomeres are highly conserved hexamer repeats (TTAGGG) at the ends of all chromosomes [1]. They maintain chromosome stability by preventing the ends from fusing to each other and allow DNA replication at chromosome ends. Telomeres are longest at birth, with the size reported to range from about 7 to 20 kb [2, 3]. The number of hexamer repeats is reduced with each round of replication, leading to its shortening from birth until end of life in somatic tissues.

The enzyme telomerase which maintains and restores the terminal regions is only expressed in embryonic/foetal cells, germ cells and other proliferating organs in adults. Short telomeres are associated with genome instability, cellular aging, some types of cancer, and several age-related diseases such as heart disease, atherosclerosis and diabetes [4, 5]. Although the rate of telomere shortening is influenced by physiological ageing and environmental factors as well, there is good evidence of genetic contribution to telomere length [6, 7].

Several methods have been developed to assess telomere length. They include procedures which quantify the mean from total DNA extracted from a population of cells, and those which measure the telomere of individual chromosomes in a cell [8]. The most established method uses southern blot hybridization analysis of terminal restriction fragments (TRF) to measure the absolute telomere length and the subtelomeric regions. However, this method is labour-intensive and time-consuming, and requires large amount of input of starting material (0.5–5 µg DNA).

With the advent of real-time polymerase chain reaction platforms, a method based on quantitative real-time PCR (qPCR) which requires about 100× less DNA, has higher throughput and faster turnaround time has become more commonly used [9]. The measurements are also more representative of telomere length as telomere primers specifically hybridise to human telomere sequences and do not depend on restriction enzyme recognition sites outside of the telomeric region which might lead to additional non-telomeric sequences being counted [10].

In this study, we tested the hypothesis that cord blood telomere length might be directly related to birth weight. Our objective was to determine the relationship between birth weight and cord blood telomere length in newborns of Chinese descent that were born in our hospital. We measured the telomere length of these cord blood samples using qPCR to assess correlation with birth weight, taking into account gestational age and maternal age.

## Methods

### Study samples

The study protocol was approved by the SingHealth Institutional Review Board which oversees all research activities in the hospital. Inclusion criteria were deliveries from mothers of Chinese descent from singleton pregnancies with no recognizable congenital disorders. Approval was granted for the anonymous collection of cord blood and limited demographic data without requiring written informed consent.

Cord blood samples were collected between March 2011 and April 2012 in our hospital, for cases that met inclusion criteria and were not involved in other research studies requiring cord blood, had not consented to donate to cord blood bank, or had not planned to store it with a private operator. Data on gestational age, gender and birth weight of the newborns, and maternal age were anonymously collected.

### Experimental details

Genomic DNA was extracted from the cord blood of 195 Chinese newborns (98 males, 97 females) using Puregene Blood Kit (Qiagen GmbH, Hilden, Germany). The quantity and quality of extracted DNA was determined by using a NanoDrop 1000 spectrophotometer (Fisher, Wilmington, DE, USA).

Telomere length assessment was done by qPCR, with *36B4* as the reference gene for normalisation using published primer sequences [9]. Amplification for both telomere copy number and the reference gene was done in separate wells but in the same experiment under the same PCR cycling conditions to reduce inter-experimental variation, in batches of five samples per experiment. The reaction mixture for *36B4* was made up of 15 ng genomic DNA, 1× Roche FastStart Universal SYBR Green Master (ROX) (Roche Diagnostics Deutschland GmbH, Mannheim, Germany), 300 nM of the forward and 500 nM of the reverse primers while the telomere reaction mix had 270 nM of the forward and 900 nM of the reverse primers. Water was added to a final volume of 20 μl. DNA samples from two healthy females were included in every experiment as internal calibration to control for inter-experimental variability. Amplification for each sample was done in quadruplicates in the Applied Biosystems StepOnePlus real time PCR system (Applied Biosystems Incorporated, Foster City, CA, USA) using the following cycling condition: 50 °C for 2 min and 95 °C for 10 min, followed by 40 cycles of denaturation at 95 °C for 15 s, and annealing-extension at 56 °C for 1 min and dissociation curve analysis.

Result was analysed using the Applied Biosystems StepOne™ software (version 2.1). C_T_ values were obtained by setting the threshold at the exponential phase of amplification. The average C_T_ values of the best three of the quadruplicates were used with one set of data discarded. Telomere copy number relative to single copy gene (T/S) ratio was calculated from the C_T_ values using the following formula: Relative T/S = 2^–(∆CT Sample − ∆CT control sample)^ = 2^−(∆∆CT)^. Where ∆C_T_^Sample^ = Sample C_T(tel)_ – Sample C_T(36B4)_ and ∆C_T_^control^ = Control C_T(tel)_ – Control C_T(36B4)_. Relative telomere length (RTL) was then obtained by comparing the T/S ratio of the test sample relative to an internal control DNA sample included in every experiment to correct for any batch variation for assays done at different times. The coefficient of variation for RTL values obtained using Control #1 is 12.7% and for Control #2 is 12.2%.

### Statistical analysis

All analyses were carried out using IBM Statistical Package for the Social Sciences version 19 (SPSS Inc., Chicago, USA). A *P* value of <0.05 was considered as statistically significant. Quantitative variables are expressed as mean and standard deviation. For each sample, RTL used was the average of the best three of the four replicates relative to each of the two control DNA samples, and gestational age-adjusted birth weight was generated by dividing the birth weight by gestational age. Correlation between individual quantitative variables was evaluated using a Pearson test. The relationship between RTL and birth weight was also explored while controlling for maternal age using partial correlation.

The newborns were placed into three groups based on clinical definition that is generally used to categorise extreme fetal growth groups (MSD Manual: http://www.msdmanuals.com/professional/pediatrics/perinatal-problems). Age-adjusted birth weight was used to classify the samples: lightest 10% (n = 20) in the small for gestational age group (SGA), heaviest 10% (n = 20) in the large for gestational age group (LGA), and the rest (n = 155) in the appropriate for gestational age group (AGA). Differences between the three groups were assessed using ANOVA for quantitative variables and Chi Square for categorical variables.

## Results

A total of 235 samples were collected from January 2011 to March 2012. Birth weight ranged from 610 to 5184 g (mean 3186.1 ± 492.4 g) and gestational age from 32 to 41 weeks (mean 38.15 ± 1.23 weeks). Of the 195 samples with DNA available for telomere length qPCR analysis, there were 97 males and 98 females. The experimentally determined telomere length showed normal distribution (Table 1). There was no statistically significant difference for all variables between male and female samples (Table 2).

**Table 1:** Characteristics of the cord blood samples analyzed

	Mean (SD)	Median	Minimum	Maximum
Mother’s age in years	32.7 (4.9)	33.0	17	47
Birth weight in grams^a^	3235 (501)	3162	1128	5184
Gestational age in weeks^a^	38.2 (1.1)	38.0	32	41
qPCR RTF (Control #1)	0.996 (0.126)	0.991	0.735	1.521
qPCR RTF (Control #2)	0.826 (0.101)	0.823	0.545	1.09
TRF (kb)	12.7 (2.5)	12.6	0.663	19.2

^a^Kolmogorov–Smirnov test of normality indicates significant deviation from normal distribution

**Table 2:** Comparison of characteristics between male and female samples

	Mean (SD)		F	*P*
	Males (n = 97)	Females (n = 98)		
Mother’s age (years)	32.7 (4.9)	32.6 (5.2)	0.015	0.902
Birth weight (grams)	3235 (501)	3158 (483)	1.204	0.274
Gestational age (weeks)	38.2 (1.1)	38.15 (1.23)	0.248	0.619
qPCR RTF/Control #1	0.989 (0.501)	1.003 (0.127)	0.551	0.459
qPCR RTF/Control #2	0.821 (0.101)	0.831 (0.101)	0.423	0.516
TRF (kb)^a^	12.4 (2.6)	12.9 (2.4)	1.617	0.205

^a^Data only available for 81 males and 78 females

Correlations between RTL and study variables were presented in Table 3. RTL values obtained using Control DNA #1 had lower standard deviation (0.007 versus 0.012 for Control DNA #2) and standard error of mean (0.00604 versus 0.00784) compared to Control DNA #2. There was highly significant correlation between the RTL from Control DNA #1 and that from Control DNA #2 (Table 3). When measured against a single copy gene, the longest RTL was almost 2× more than the shortest when calibrated against both Control DNA #1 (range 0.5445–1.0860; SD: 0.1007) and Control DNA #2 (range 0.7351–1.5210; SD: 0.1261).

**Table 3:** Correlation analysis of RTL with different variables

	RTL (Control #1)		RTL (Control #2)	
	Pearson’s R	*P* (2-tailed)	Pearson’s R	*P* (2-tailed)
TRF	0.144	0.069	0.195	0.014*
Maternal age	0.001	0.989	−0.003	0.964
Gestational age	0.108	0.131	0.012	0.863
Birth weight (BW)	0.201	0.005*	0.158	0.028*
Age-adjusted BW	0.199	0.005*	0.172	0.017*
Control DNA #2 (or #1)	0.801	0.000*	0.801	0.000*

* Statistically significant

**Fig. 1:**
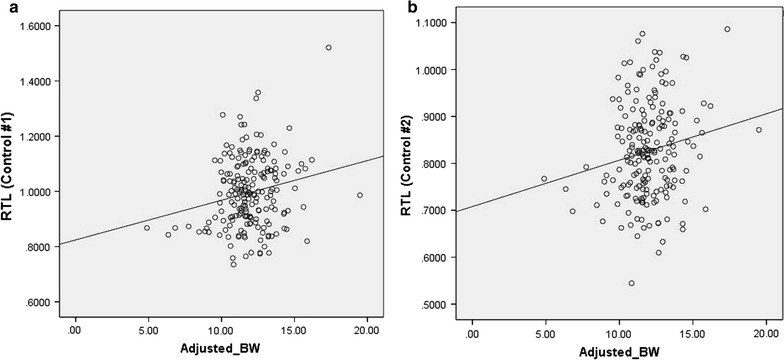
Correlation of RTL with age-adjusted birth weight **a** using Control #1 (Pearson correlation = 0.198, *P* = 0.005) and **b** using Control #2 (Pearson correlation = 0.171, *P* = 0.017)

There was a statistically significant correlation of RTL with birth weight, with the heavier newborns having longer RTL (Fig. 1; Table 3). There was also correlation of RTL with TRF data obtained previously [3] for these samples (Table 3). However, there was no statistically significant correlation with maternal age and gestational age. Adjusting for maternal age did not result in any difference in statistical significance.

**Fig. 2:**
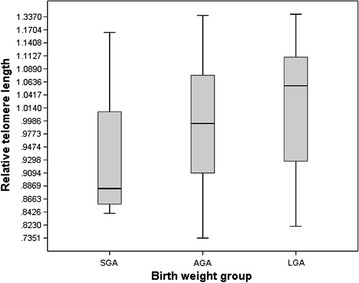
Mean RTL for the three birth weight groups based on clinical definition of birth weight (SGA/AGA/LGA) using results from Control #1 (*P* = 0.022)

Analysis using tertile birth weight groups and different models (linear, quadratic, cubic, polynomial) did not reveal any statistically significant correlation. The extreme group design was then used to classify the newborns into three groups based on age-adjusted birth weight: lightest 10% (Group SGA), heaviest 10% (Group LGA), and the rest (Group AGA). There were statistically significant differences among the groups in terms of RTL (*P* = 0.022) normalised to Control #1 (Fig. 2a). Group SGA had a mean RTL of 0.7922 compared to 0.8537 for Group LGA (*P* = 0.037), with the mean for Group AGA at 0.8270 (*P* = 0.050). The difference between Group AGA and Group LGA was not statistically significant.

There was a statistically significant difference (*P* = 0.009) in mean gestational age for the three groups: with the mean for SGA at 37.30 ± 2.27 weeks, AGA at 38.24 ± 1.03 and LGA at 38.30 ± 0.865. The median for all three groups were the same at 38.00 weeks. There was no statistically significant difference between the three groups in the mean for maternal age (*P* = 0.653) or TRF measured previously (*P* = 0.332).

Eight cases were from preterm deliveries of between 32 and 36 weeks (mean gestational age 34.6 ± 1.69 weeks). Although the preterm group has shorter RTL of 0.933 ± 0.139 compared to the 0.998 ± 0.125 for the full-term group (gestational age ≥36 weeks; mean 38.3 ± 0.952 weeks), the difference was not statistically significant (*P* = 0.153). There was also no statistically significant difference in the mean maternal age between the preterm and full-term birth groups (*P* = 0.911).

## Discussion

It has been shown that the DNA extraction method used can affect telomere length measurement due to differences in purity and integrity [11, 12]. In this study, all the samples were extracted using the same method. The RTL results are largely consistent between experiments and also between different reference control DNA samples.

Telomere length in this study cohort had been determined by TRF previously [3], and was measured by qPCR as a comparison in the current study. Similar to other recent studies [13, 14], there was only moderate correlation between telomere length measured by qPCR and TRF. Our correlation appears to be lower than some of the published data. One explanation is that our population on the whole was more polymorphic at the subtelomeric region restriction enzyme digestion sites used in TRF analysis, which might have resulted in higher values for telomere length for some samples [15, 16].

Our result of shorter telomeres in lower birth weight newborns is consistent with the reported reduced telomere length in intrauterine growth restriction (IUGR) placentas compared to controls as measured by quantitative FISH [17, 18]. Our sample size is larger than most previous studies, with all the samples obtained from a single ethnic group. Our data showed direct correlation of both unadjusted and gestational age-adjusted birth weight with relative telomere length.

Additional analysis using tertile birth weight groups and different models did not reveal any statistically significant correlation. Conversely, there is a statistically significant difference when the newborns are divided into three groups based on gestational age-adjusted birth weight, with the shortest telomeres found in the group with the lowest birth weight. This extreme group method of categorizing birth weight (SGA/AGA/LGA) is typically used by studies investigating the association of telomere length with birth parameters. It is based on clinical relevance as both LGA and SGA groups are at higher risk of maternal health complications, adverse neonatal outcomes and future health morbidity. LGA newborns are at increased risk of birth injuries during deliveries. They may have polycythemia, hypoglycemia and immature lungs if the mothers are diabetic, and increased risk of diabetes and obesity in adulthood [19]. However, the risk for these later life disorders could also be due to genetic predisposition if their mothers were predisposed to gestational diabetes during the pregnancies.

For SGA neonates and in particular, those with intrauterine growth restriction (IUGR), there is an increased risk of cardiovascular and metabolic diseases [20]. Some of the diseases are also those which have been reported to be associated with short telomeres. Shorter cord blood telomere length has also been associated with perinatal outcomes such as gestational diabetes and preterm premature rupture of membranes [21, 22]. The risk for those born as SGA might be related to being exposed to unfavorable in utero conditions that lead to oxidative stress and high turnover of cells during the embryonic period, both of which are known to lead to reduced telomere length. The unfavorable growth might also predispose them to the development of adult diseases such as hypertension, cardiovascular disease, and type 2 diabetes [23]. As some IUGR newborns are known to have conditions linked to aged phenotypes such as chronic hypertension and hyperglycemia, it is not implausible that their telomeres might also showed some “ageing” and appear shorter than age-matched controls with normal birth weight. Experimental evidence from a study subjecting lymphocyte cultures derived from cord blood samples to a genotoxic agent showed that those with shorter telomeres had more genetic damage as measured by the frequency of binucleated cells with micronuclei [24].

A recent study (N = 69) found an inverse correlation between telomere length and birth weight groups, with the LGA group having the shortest telomeres and the SGA group having the longest compared to their average weight counterparts [25]. In our study cohort, telomere length for the top 10% in terms of age-adjusted birth weight was longer and not shorter than the other two groups, while the bottom 10% had the shortest telomeres. Our results are in agreement with two other recent studies (N = 54 and 103) using qPCR on cord blood DNA, both reporting that telomere length at birth was longer in larger newborns [26, 27]. Another study (N = 66) found no association with birth weight but the data on telomere length for specific birth weight categories was not presented [28]. Similar to our data, they did not find statistically significant correlation between gestational age and telomere length. Using a different design, a group reported significant positive correlation between intra-pair differences of birth weight and telomere length for monozygotic but not dizygotic twin pairs, and that monozygotic twins with the larger difference in birth weight also have larger difference in telomere length [29]. These results suggest that the in utero environment which affect foetal growth also influences telomere length, and that TL is partly determined by the genetic background as the correlation is only statistically significant in monozygotic twin pairs. Inter-individual genetic variation could explain why some studies (especially those with small samples sizes) did not show correlation with birth weight, as the genetic factors influencing telomere length are largely unknown and thus could not be measured at present.

We previously reported that females have longer telomeres as measured by TRF. The data for qPCR RTL showed a similar trend although the difference was not statistically significant (Table 2). Recently, Wojcicki et al. also reported shorter telomere length at birth for males among Latino infants [27].

There are several limitations of this study. We do not have the clinical data on the mothers who might have medical conditions that impacted the growth of their foetuses; our data on birth weight and gestational age was collected anonymously and the mothers’ pre-pregnancy weight or body mass index was not available for analysis. We also did not have the DNA from the parents for parental RTL measurement for correlation or adjustment of the newborns’ RTL data. In addition, the qPCR method measures the average TL of all chromosomes in multiple cells, which cannot distinguish whether some cells or chromosomes have some telomeres that are significantly shorter or longer than the rest.

In summary, our RTL results using a medium sized cohort from a single ethnic group show a direct correlation of birth weight with mean qPCR telomere length which measures the true length of the telomere hexamers. As telomere length is a reflection of replicative history, the shorter telomeres could be a reflection of higher cellular turnover during the embryonic period for the newborns that had suboptimal growth. Future work could look into whether there might be a subpopulation of cells with shorter telomeres. The presence of individual chromosomes with much shorter telomeres can be explored using more sensitive methods that directly measure telomere length at the resolution of a single chromosome end.

## Abbreviations

AGA: appropriate for gestational age
FISH: fluorescent in situ hybridization
IUGR: intrauterine growth restriction
LGA: large for gestational age
qPCR: quantitative polymerase chain reaction
RTL: relative telomere length
SGA: small for gestational age
TRF: terminal restriction fragments
